# Biodetector for chlordane using doped InP_3_ monolayers: a density functional theory study

**DOI:** 10.1039/d4ra06303a

**Published:** 2024-10-25

**Authors:** Yui Mary Mei, Xuan Luo

**Affiliations:** a National Graphene Research and Development Center Springfield Virginia 22151 USA

## Abstract

Chlordane is a serious pollutant in the environment, and it is necessary to monitor chlordane levels using biodetectors. We performed first-principles calculations to investigate the adsorption of chlordane on Ag, Pd, and Au doped InP_3_ semiconductor monolayers. The results indicated that the adsorption energies of chlordane adsorbed on Ag, Pd, and Au doped InP_3_ are −7.961 eV, −6.328 eV, and −7.889 eV respectively. The band gaps of the doped InP_3_ monolayers underwent drastic changes after coming in contact with chlordane, the largest change in band gap occurred for Pd-doped InP_3_, where the band gap changed from 0.024 eV to 0.335 eV. The large change in band gap shows that the monolayer is sensitive to the molecule, making it a good biodetector. Our results conclude that Pd-doped InP_3_ stands out as the most promising biodetector for chlordane. This result will benefit environmental experimentalists in their further research.

## Abstract 2

Chlordane is a hazard to the environment and using doped InP_3_ monolayers as biodetectors is a good solution.

## Graphical abstract

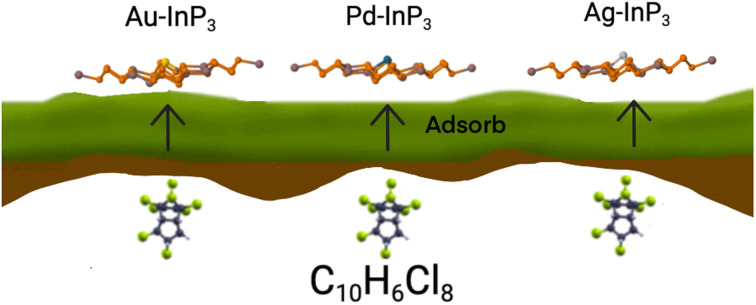

## I. Introduction

Pollution, with its far-reaching impacts on soil and water quality, poses a critical threat to public health and ecosystems worldwide. A common type of pollutant called persistent organic pollutants (POPs) has quickly become a global concern because of its detrimental effects on soil and water quality.^1^ There are three main types of POPs, the first being industrial materials such as polychlorinated biphenyls (PCBs), industrial waste such as polyaromatic hydrocarbons (PAHs), and organochlorine pesticides (OCPs).^2^ Organochlorine pesticides are synthetic pesticides that were widely used to control insect pests in agriculture and forestry all around the world.^3^ OCPs are banned in many parts of the world,^3^ classified as a type of neurotoxin,^4^ and an EDC (endocrine disrupting chemical)^5^ and can pose serious problems to one's health. Consequences of short-term exposure to these pesticides include convulsions, nausea, and tremors.^6^ Long-term exposure can cause an increased risk of dementia or Parkinson's disease;^7^ increased risk of certain cancers;^8^ and damage to the endocrine system.^9^ Although OCPs were banned decades ago, some remnants remain in the environment due to the persistent nature of the pesticides.^10^ A variety of such pesticides exist, some common examples include dichlorodiphenyltrichloroethane (DDT) for mosquito control,^11^ endosulfan for fruit fly and larvae control,^12^ and chlordane for termite control in homes.^13^ Chlordane was the second most important organochlorine insecticide, had an estimated annual production of nine million kilograms, and was used in millions of homes annually.^14^ It physically presents as a thick, colourless or amber liquid, has a mild but irritating smell,^15^ and a chemical formula of C_10_H_6_Cl_8_. It was banned in 1988 (ref. 16) but was used in lawns, homes, and agricultural fields as a form of pesticide and termite control.^17^ Chlordane is still a concern due to its ability to stick to soil,^18^ low solubility in water,^17^ and lipophilicity.^19^ It can still be found in treated areas, agricultural run-off water, and in the dust of buildings where it was once used.^20^

Previous studies on chlordane covered a variety of topics, some examples being: its accumulation in people and animals as well as its persistence in nature. An earlier study on the accumulation of technical chlordane in fish and wildlife found that a concentration between 36.9–59 μg per 1 specimen is enough to cause a 50 percent mortality rate, concluding that chlordane is highly toxic.^21^ Oloff *et al.* conducted a study on OCP solubility and accumulation in nature and found that chlordane does not show any signs of metabolic breakdown over a 12 weeks period, demonstrating its danger to ecosystems.^22^ A study on the effects of chlordane on the human and rat liver concluded that chlordane caused an increase in levels of triglycerides (TG), creatine phosphokinase (CPK) and lactate dehydrogenase (LDH) as well as an increase in liver weight in both humans and rats.^23^ Parada *et al.* investigated chlordane in relation to breast cancer survival and concluded that chlordane not only likely increases one's risk of cancer but also reduces one's survival rate.^24^ Although there are many studies on the negative effects of chlordane on both living organisms and the environment, there are few studies on the detection and adsorption of chlordane to mitigate these risks.

A previous study by Qin *et al.*^25^ explored the detection and adsorption of different OCPs from the environment using a pure InP_3_ monolayer, but chlordane was not a part of this study. The lack of previous studies is evidence that the problem of chlordane pollution is often overlooked. However, it is important to be able to detect and absorb chlordane, a toxic chemical, from the environment for safety. One of the leading ways to do this is by using semiconductor monolayers, as shown from previous research.^26,27^ A semiconductor monolayer is a type of material that is a single molecule/atom in thickness made of a semiconductor.^28^ Some common examples of semiconductor monolayers include: graphene,^29^ MXene group,^30^ and transition metal dichalcogenides (TMDs).^31^ An example of a group of semiconductor monolayers that may interact with chlordane is the aforementioned MXene group because they can conduct electricity^32^ and are highly biocompatible.^33^ MXenes are a group of 2D transition metal carbide/nitride with a composition of M_*n*+1_X_*n*_T_*x*_, where M is a transition metal, X is nitrogen or carbon, and T is a surface functional group.^34^ However, more promising candidates for the detection and adsorption of chlordane are the allotropes SnP_3_,^35^ GeP_3_,^36^ and InP_3_ (ref. 37) as shown from previous research. Specifically, the monolayer InP_3_ is of great interest in being a sensitive detector for chlordane. Some of the previous applications of InP_3_ are in lithium batteries,^38^ the detection of various chemicals such as formaldehyde^39^ and *SF*_6_,^40^ and the detection of certain organochlorine pesticides.^25^

One of the many methods of detection and adsorption for organochlorine pesticides is using doped semiconductor monolayers.^41^ For chlordane specifically, InP_3_ (indium triphosphide) is a promising candidate because of its graphene-like structure, high electron mobility, chemical stability and adjustable band gap.^42^ Furthermore, the In–P bond also shows strong attraction to select polar molecules because of its polar covalent nature, making it a sensitive detector.^25^ A graphene-like structure is especially beneficial for sensing because of its excellent adsorption property,^43^ high conductivity of both heat and electricity^44^ and large specific surface area.^45^ A large specific surface area means that there is a high density of receptors in a given area, making the material sensitive. To optimize InP_3_ for chlordane detection, its band gaps are adjusted using palladium (Pd), gold (Au) and Silver (Ag) based on previous research. Palladium was chosen based on previous research showing improved sensitivity to CO, NH_3_, O_2_ and NO_2_ when doped into graphene.^46,47^ This is important as the chemical formula of chlordane is C_10_H_6_Cl_8_, so it is vital that the modified InP_3_ monolayer is sensitive to its elements C, H and Cl. Next, gold was selected based on previous research demonstrating its affinity for modifying monolayers to be more sensitive to chlorine.^48^ Silver was selected due to its previously shown ability to modify InP_3_ to be more sensitive to NO_2_.^47^ This study hopes that by modifying InP_3_, the detection and adsorption of chlordane can be more effective.

The density functional theory (DFT) is used in this study to analyze the effectiveness of each dopant (Ag, Pd, Au) in modifying the InP_3_ monolayer for detecting chlordane (C_10_H_6_Cl_8_). Calculations were conducted to find the optimal electronic and atomic structure of each complex. The results were then analyzed and the different monolayers were compared to find the most optimal for detecting chlordane.

## II. Method

### A. Computational details

The density functional theory (DFT) incorporated into the ABINIT package^49^ implements the generalized gradient approximation (GGA)^50^ exchange–correlation functionals with a Perdew–Burke–Ernzerhof (PBE) format. The projected augmented wave (PAW) method^51^ is used to produce pseudopotentials using the AtomPAW^52^ code. The electron configurations of hydrogen (H), carbon (C), phosphorus (P), chlorine (Cl), palladium (Pd), silver (Ag), indium (In), and gold (Au) are shown in Table 1.

**Table 1:** Electron configurations and radius cutoffs of elements used in current research

Element	Configuration	Radius cutoff (Bohrs)
Hydrogen (H)	1s^1^	1.00
Carbon (C)	[He]2s^2^2p^2^	1.32
Phosphorus (P)	[Ne]3s^2^3p^3^	1.85
Chlorine (Cl)	[Ne]3s^2^3p^5^	1.49
Palladium (Pd)	[Kr]4d^10^	3.19
Silver (Ag)	[Kr]4d^10^5s^1^	3.12
Indium (In)	[Kr]4d^10^5s^2^5p^1^	2.95
Gold (Au)	[Xe]6s^1^4f^14^5d^10^	3.29

### B. Convergence details

In the total energy calculation, the self-consistent field (SCF) cycle stopped once the total energy difference was less than 1.0 × 10^−10^ Ha twice consecutively. The convergence of kinetic energy cutoff, Monkhorst–Pack^53^*k*-point grids, and vacuum layer was also calculated. The convergence criterion was fulfilled when the total energy difference of datasets was smaller than 0.0001 Ha (0.003 eV) twice consecutively.

Using the Broyden–Fletcher–Goldfarb–Shanno minimization (BFGS)^54^ structural optimization of the chlordane molecule, pure indium triphosphide monolayer, Au-, Pd-, Ag-doped monolayers, and their complex systems were performed. The tolerance for the maximum force of each atom is less than 2.0 × 10^−3^ Ha/Bohr (0.01 eV Å^−1^). The SCF cycle will be terminated once the force difference is smaller than 1.0 × 10^−10^ Ha/Bohr (0.01 eV Å^−1^) twice consecutively.

### C. Atomic structure

The structure of *cis*-chlordane, with a chemical formula of C_10_H_6_Cl_8_ was optimized along with the primitive cell of InP_3_. The process was repeated with the 3 × 3 × 1 monolayer InP_3_ with 32 total atoms (8In, 24P) and interstitial doping of the monolayer by placing the dopant (an atom of Ag, Pd, and Au) in the center of the monolayer – its most optimal spot - created the doped 3 × 3 × 1 supercell. The substrates are then optimized according to the aforementioned convergence requirements. The defect formation energy (given by *E*_f_) of an atom on InP_3_ is given by

1 *E*_f_ = *E*_Dopant/monolayer_ − *E*_monolayer_ − *E*_Dopant_

where *E*_Dopant/monolayer_ is the total energy of the monolayer, *E*_monolayer_ is the total energy of the 3 × 3 × 1 InP_3_ monolayer, and *E*_Dopant_ is the total energy of the pure element.^55^ Next, chlordane was placed on top of the optimized monolayers in a manner that maximized the amount of chlorine interaction with the monolayer/dopant.

### D. Electronic structure

#### 1. Band structure

The band structures were computed along the high symmetry *k*-points *Γ* (0, 0, 0), *M* (1/2, 0, 0), *K* (1/3, 2/3, 0), and *Γ* (0, 0, 0). The converged charge density values of optimized structures of the monolayer (pure and doped with Ag, Pd, Au) were used to calculate the band structure. Next, a similar process is repeated for the complex system of chlordane placed on the different monolayers.

#### 2. Projected density of state

To analyze chlordane adsorption on pure, Ag-doped, Pd-doped, and Au-doped monolayers, calculations of the projected density of states (PDOS) were conducted using the tetrahedron method. The atoms for the calculations were selected based on their proximity to the gas exchange site.

#### 3. Charge transfer

Furthermore, the interactions between chlordane and the different substrates are further analyzed by calculating the charge transfer between the atoms. The equation for charge transfer is represented as

2 Δ*ρ* = *ρ*_molecule/monolayer_ − *ρ*_monolayer_ − *ρ*_molelcule_

where Δ*ρ* represents the net charge transfer and *ρ*_molecule/monolayer_, *ρ*_monolayer_, *ρ*_molecule_ represent the charge density of the chlordane-substrate system, the substrate, and the molecule respectively.^56^

#### 4. Biodetector sensability

Sensing response is vital for a biodetector and can be calculated with the equation3
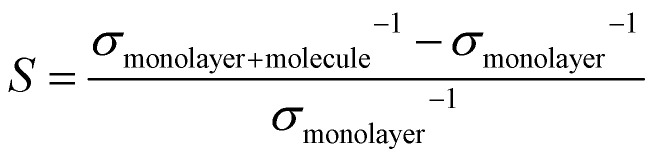
where *S* is the sensing response, *σ*_monolayer*+*molecule_ and *σ*_monolyer_ indicate the conductivity of the chlordane + monolayer and the monolayer system respectively.^57^ The electrical conductivity (*σ*) of the InP_3_ systems can be calculated with the equation:

4 *σ* ∝ exp(−*E*_g_/2*k*_B_*T*)

where *E*_g_, *k*_B_, and *T* represent the band gap of the different substrates, the Boltzmann constant, and the temperature (room temperature is 300 K) respectively.^58^

### E. Adsorption calculations

The optimized chlordane structure was placed on top of the various prepared monolayers and the adsorption energies were calculated:

5 *E*_ad_ = *E*_molecule+monolayer_ − *E*_monolayer_ − *E*_molecule_

in which *E*_ad_, *E*_mol+monolayer_, *E*_monolayer_, and *E*_mol_ represent the adsorption energy, the total energy of chlordane added to the total energy of the InP_3_ monolayer system, InP_3_ monolayer, and the chlordane molecule, respectively.^55^

## III. Results

**Fig. 1:**
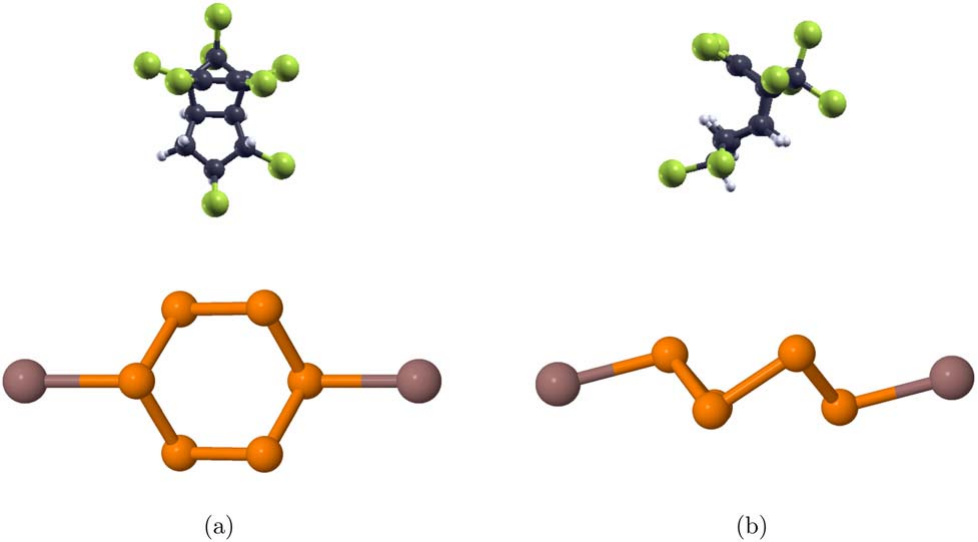
Optimized structures of chlordane (a) top view of chlordane molecule and InP_3_ primitive cell (b) side view of chlordane molecule and InP_3_ primitive cell. Navy, white, green, orange, and brown colors are used to represent carbon, hydrogen, chlorine, phosphorus, and indium respectively.

**Fig. 2:**
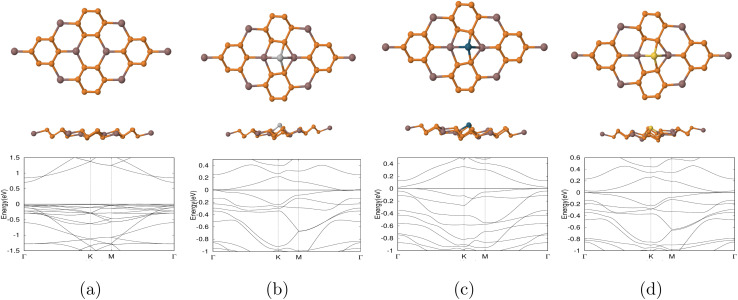
Optimized structures of top and side view of (a) pure 3 × 3 × 1 InP_3_ monolayer (b) Ag-doped 3 × 3 × 1 InP_3_ monolayer (c) Pd-doped 3 × 3 × 1 InP_3_ monolayer (d) Au-doped 3 × 3 × 1 InP_3_ monolayer. Orange, brown, silver, blue, and gold, represent phosphorus, indium, silver, palladium, and gold respectively.

The structural and adsorption properties before and after the adsorption of chlordane molecule onto the pure InP_3_ monolayer were analyzed first to use as *a* point of reference. Next, Ag, Pd, and Au are interstitially doped onto the pure monolayer to improve the adsorption of chlordane into InP_3_. Then, the adsorption energy, band structure, sensibility, PDOS, and charge transfer of the doped monolayers were calculated to assess their affinity for detecting and absorbing chlordane.The optimized structures of chlordane (C_10_H_6_Cl_8_) and InP_3_ primitive cell are shown in Fig. 1. The calculated structural parameters are shown in Table 2 and are in good accordance with other theoretical studies.^55,59^To find the most optimal structure of InP_3_ substrate, 2 × 2, 3 × 3, and 4 × 4 supercells were tested and it was found that the 3 × 3 supercell was optimal according to the aforementioned convergence requirements. Using the optimized atomic configurations, the band structures of the 3 × 3 pristine and doped InP_3_ are calculated and presented in Fig. 2 and Table 3. Pristine InP_3_ has an indirect band gap of 0.703 eV, Ag doped InP_3_ has a band gap of 0, Pd doped InP_3_ has a direct band gap of 0.024 eV, and Au doped InP_3_ has an indirect band gap of 0.013 eV.

**Table 2:** Calculated and experimental results for the structural parameters of the chlordane molecule and the InP_3_ monolayer

	Bond lengths and bond angles	Current study (Å)	Other study (Å)
C_10_H_6_Cl_8_	C–Cl	1.76	1.84 (ref. 59)
	C–H	1.10	
	C–C (double bond)	1.57	
	C–C (single bond)	1.54	
	*∠* C–C–C	108.2°	117 (ref. 59)
	*∠* Cl–C–Cl	107.9°	107.2 (ref. 59)
InP_3_	P–P	2.24	2.24 (ref. 55)
	In–P	2.57	2.53 (ref. 55)
	*∠* P–in–P	113.3°	113.3° (ref. 55)
	*∠* P–P–P	92.8°	92.2° (ref. 55)

**Table 3:** Optimized structural parameters for pure and doped 3 × 3 × 1 InP_3_ monolayers: bond lengths (d), lattice constant (a), band gap (*E*_g_), band gap after chlordane contact (c), and deformation energy (*E*_f_)

Substrate	*d* (Å)	*a* (Å)	*E*_g_ (eV)	*E*_f_ (eV)
InP_3_	—	28.5019	0.703	0.25267
Ag-doped InP_3_	Ag–P 2.64	28.461	0	0.253
	Ag–In 2.99			
Pd-doped InP_3_	Pd–P 2.35	28.362	0.024	0.342
	Pd–In 2.85			
Au-doped InP_3_	Au–P 2.5	28.463	0.013	0.336
	Au–In 2.95			

**Fig. 3:**
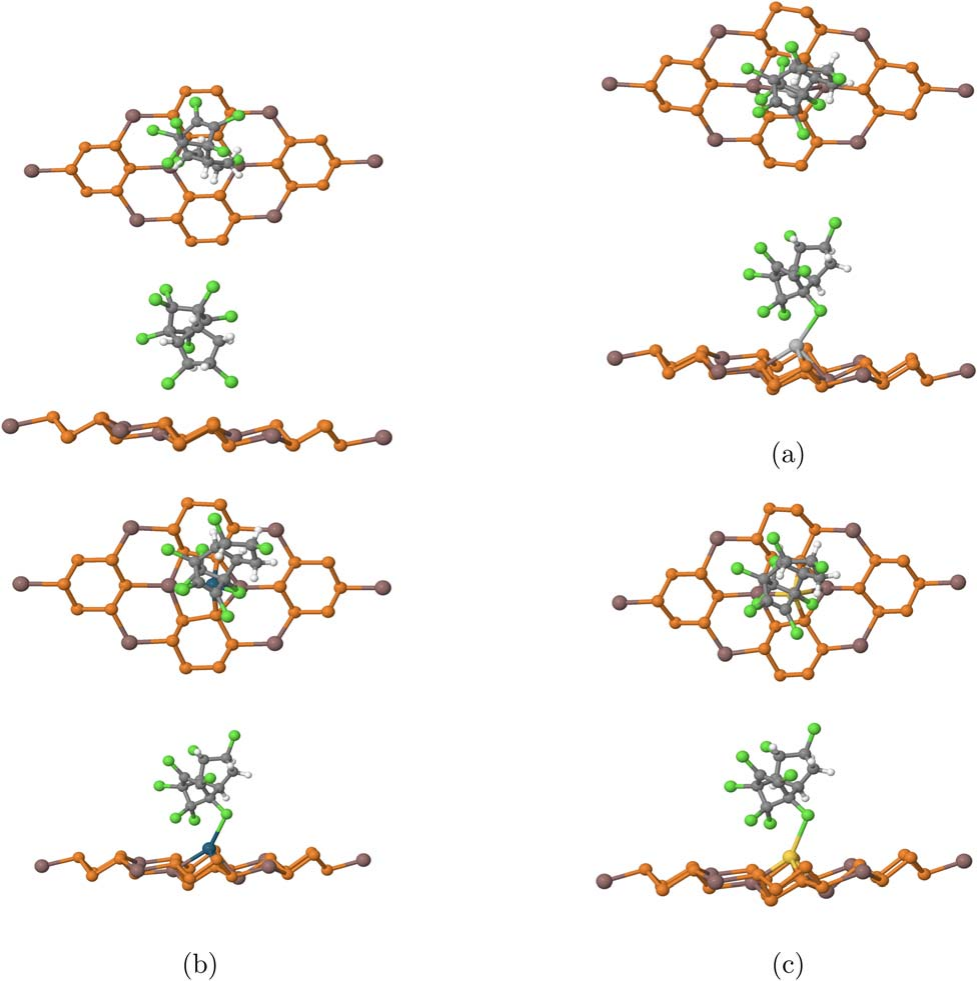
Optimized structures of top and side view of the systems investigated in this study: chlordane placed on top of (a) pure InP_3_ monolayer (b) Ag-doped InP_3_ monolayer (c) Pd-doped InP_3_ monolayer (d) Au-doped InP_3_ monolayer. Gray, white, green, orange, brown, light gray, blue, and gold, represent carbon, hydrogen, chlorine, phosphorus, indium, silver, palladium, and gold respectively.

Chlordane was oriented and placed centrally on top of the substrate in a manner that guarantees as many as possible interactions between its Cl atoms and the substrate's dopant and indium atoms. It is known that InP_3_ interacts well with polar molecules such as chlordane. By orienting chlordane so that the maximum amount of chlorine atoms (3) is facing toward the substrate, chlorine can more easily interact with the metal dopants and indium. Note that a chemical reaction does not occur as the dissociation energy for chlordane bonds is larger than the calculated adsorption energies (*E*_ad_). After relaxation, the adsorption energy (*E*_ad_), bond length between Cl and the dopant, and band gap (*E*_g_) for each complex system are calculated and shown in Table 4. The relaxed structures are shown in Fig. 3. A positive adsorption energy indicates an endothermic reaction with a non-spontaneous adsorption while a negative adsorption energy indicates an exothermic reaction with a spontaneous adsorption.^47^ The more negative the adsorption energy is, the stronger the interaction between chlordane and InP_3_ substrates would be.^47^ The comparatively less negative adsorption energy value (eV) for the interaction between chlordane and pristine InP_3_ indicates a weaker interaction between the two, signifying that pristine InP_3_ would not make a great detector for chlordane. Comparatively, a smaller adsorption energy value between Ag-doped InP_3_, Au-doped InP_3_ and chlordane suggests that both have strong interactions with chlordane while Pd-doped InP_3_ have a slightly weaker interaction with chlordane given its less negative adsorption value.To determine if a material is sensitive to a molecule, the change in band gap (before/after coming in contact with the molecule) must be considered. The most significant change in band gap is seen in Pd doped InP_3_ where the gap changed from 0.024 eV to 0.335 eV, likely suggesting that Pd doped InP_3_ is sensitive to chlordane. Next, the band gap of Au-doped InP_3_ changed from 0.013 eV to 0, signifying that after coming in contact with chlordane, the material may have become metallic. A metallic material is also extremely good for sensing of its high conductivity, which when used in eqn (3) means that the material is sensitive to the compound. However, the band gap for Ag-doped InP_3_ remains unchanged from 0 to 0, and it is impossible to know for certain if the material is sensitive to chlordane. Pristine InP_3_ underwent very little change after coming in contact with chlordane and the structure remains unchanged, suggesting limited chlordane sensitivity. The change in band gaps before and after contact with chlordane is shown in Table 5.

**Table 4:** The adsorption energy (*E*_ad_), bond length between chlordane and dopant (d), band gap (*E*_g_) between chlordane and the substrates

System	*E*_ad_ (eV)	*d* (Å)	*E*_g_ (eV)
InP_3_	−0.065	—	0.683
Ag-doped InP_3_	−7.961	2.970	0
Pd-doped InP_3_	−6.328	2.350	0.335
Au-doped InP_3_	−7.889	3.18	0

**Table 5:** The band gaps of the pure and doped InP_3_ monolayers before and after interacting with chlordane

System	Band gap before chlordane (*E*_*g*_) (eV)	Band gap after chlordane (*E*_*g*_) (eV)
InP_3_	0.703	0.683
Ag-doped InP_3_	0	0
Pd-doped InP_3_	0.024	0.335
Au-doped InP_3_	0.013	0

**Fig. 4:**
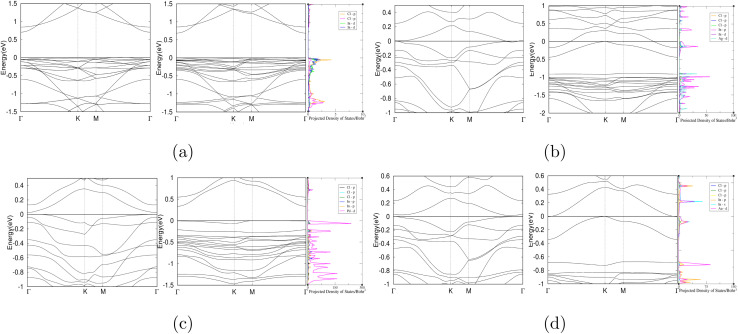
Band structures and PDoS graphs of chlordane on (a) pure InP_3_ monolayer (b) Ag-doped InP_3_ monolayer (c) Pd-doped InP_3_ monolayer (d) Au-doped InP_3_ monolayer. Refer to the legend on each individual PDoS graph for the representation of the different atoms and orbitals.

**Fig. 5:**
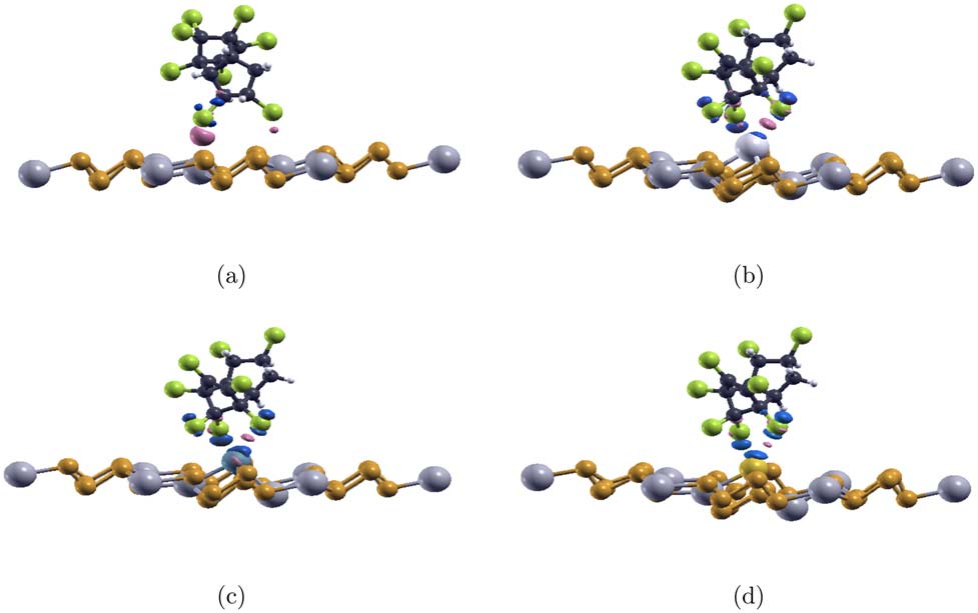
Charge transfer of chlordane on (a) pure InP_3_ (b) Ag-doped InP_3_ (c) Pd-doped InP_3_ (d) Au-doped InP_3_. The pink areas indicate electron accumulation, while the blue regions show electron depletion. The gray, orange, green, navy blue, white, light gray, turquoise, and yellow colors represent In, P, Cl, C, H, Ag. Pd, Au, respectively. The isovalue is set at 0.015 electron/Bohr3.

The projected density of states (PDoS) for each complex was graphed and shown in Fig. 4. For the pristine InP_3_ notable hybridization of the orbitals occurs below Fermi level at 0.005 eV, and 1.28 eV, marking 2 hybridization points. Comparatively, notable hybridization occurs at many more points for all of the other substrates. Specifically, for Ag-doped InP_3_ significant hybridization occurs below Fermi level at 0.2 eV, 0.9 eV, and 1.01 eV; Au-doped InP_3_ shows hybridization at 0.21 eV and below Fermi level at 0.1 eV and 0.7 eV; and Pd-doped InP_3_ shows hybridization at −0.01 eV, −0.3 eV, and −0.7 eV. The numerous hybridization sites of the different orbitals across all the graphs indicate a strong interaction between chlordane and the various doped substrates. Obviously, pristine InP_3_ has less hybridization with the chlordane molecule as shown in Fig. 5a aligns with the previous evidence that pristine InP_3_ is not the optimal detector for chlordane. The calculated PDoS results are in strong agreement with the band structures and adsorption energy calculations.

Using eqn (2), the charge transfer isosurfaces between the chlordane molecule and substrates are shown in Fig. 5. Electrons deplete around the dopant and accumulate around the chlorine atoms. Comparatively, there is a slightly greater electron exchange between the Pd-doped InP_3_ and chlordane than the other doped substrates. Notably, the pristine InP_3_ has very little electron transfer between the chlordane molecule, aligning with previous conclusions that pristine InP_3_ is not a good detector for chlordane. Although all three doped InP_3_ substrates demonstrate strong chemisorption, pristine InP_3_ shows physisorption which further supports conclusions that pristine InP_3_ is not a great detector for chlordane.

## IV. Conclusion

In summary, pristine and doped InP_3_ were investigated using density functional theory to explore the most optimal detector for chlordane. We calculated the adsorption energy, band structure, projected density of states, and charge difference before and after chlordane adsorption.

While all 3 doped monolayers are capable of adsorbing chlordane, Ag doped InP_3_ has the best adsorption value at −7.961 eV and pure InP_3_ has the worst adsorption value at −0.065 eV. The greatest band gap change occurred for Pd doped InP_3_ where it changed from 0.024 eV to 0.335 eV after interacting with chlordane. Given Pd doped InP_3_'s good adsorption value at −6.328 eV and its large change in band gap, it is the most promising substrate.

## Data availbility

Data for this article, including the PAW datasets used are available on the ABINIT website at https://www.abinit.org/. The code for ABINIT can be found at https://abinit.github.io/abinit_web/download.html with DOI-https://doi.org/10.1016/j.cpc.2019.107042. The version of the code employed for this study is version 10.0.7. The ATOMPAW code used to generate the pseudopotentials can be found at https://github.com/atompaw with DOI-https://doi.org/10.1016/S0010-4655(00)00244-7 The XCrySDen program used to process figures can be found at http://www.xcrysden.org/Download.html with DOI-https://doi.org/10.1016/S1093-3263(99)00028-5.

## Conflicts of interest

There are no conflicts to declare.
